# Auditory hair cell defects as potential cause for sensorineural deafness in Wolf-Hirschhorn syndrome

**DOI:** 10.1242/dmm.019547

**Published:** 2015-09-01

**Authors:** Mohi Ahmed, Kiyoe Ura, Andrea Streit

**Affiliations:** 1 Department of Craniofacial Development and Stem Cell Biology, King's College London, London, SE1 9RT, UK; 2 Laboratory of Chromatin Metabolism and Epigenetics, Graduate School of Science, Chiba University, 1-33, Yayoi-cho, Inage-ku, Chiba 263-8522, Japan; 3 PRESTO, Japan Science and Technology Agency (JST), Kawaguchi, Saitama 332-0012, Japan

**Keywords:** Deafness, Ear, Hair cells, Histone modification, Methyltransferase, Mouse, Stereocilia

## Abstract

WHSC1 is a histone methyltransferase (HMT) that catalyses the addition of methyl groups to lysine 36 on histone 3. In humans, *WHSC1* haploinsufficiency is associated with all known cases of Wolf-Hirschhorn syndrome (WHS). The cardinal feature of WHS is a craniofacial dysmorphism, which is accompanied by sensorineural hearing loss in 15% of individuals with WHS. Here, we show that *WHSC1*-deficient mice display craniofacial defects that overlap with WHS, including cochlea anomalies. Although auditory hair cells are specified normally, their stereocilia hair bundles required for sound perception fail to develop the appropriate morphology. Furthermore, the orientation and cellular organisation of cochlear hair cells and their innervation are defective. These findings identify, for the first time, the likely cause of sensorineural hearing loss in individuals with WHS.

## Abstract (precis)

**Editor’s Choice:** WHS is associated with sensorineural deafness. Here, the authors show that, although cochlear hair cells are specified normally in a WHS mouse model, they are disorganised and display sterocilia defects.

## INTRODUCTION

Epigenetic processes, including histone modifications by specific enzymes, are crucial for controlling gene expression, but are often altered in various cancers and other disease conditions. One such histone-modifying enzyme is Wolf-Hirschhorn syndrome candidate 1, *WHSC1* (also known as *NSD2* and *MMSET*), mutations in which cause cancer and the human disorder, Wolf-Hirschhorn syndrome (WHS; OMIM 194190). WHS is a rare condition with an estimated prevalence of 1/20,000 to 1/50,000 births and a female predilection of 2:1 (Battaglia et al., 1999; Bergemann et al., 2005; Maas et al., 2008). It is caused by the deletion of two WHS critical regions – WHSCR-1 (Wright et al., 1997) and WHSCR-2 (Zollino et al., 2003). WHSCR-1 is located ∼2 Mb away from the end of chromosome 4p16.3 and contains two genes in close proximity, *WHSC1* and *WHSC2* (Wright et al., 1997). WHSCR-2 is located between 1.4 and 1.9 Mb away from the end of 4p16.3 and the locus contains a number of genes, including fibroblast growth factor receptor 3 (*FGFR3*), leucine zipper-EF-hand containing transmembrane protein 1 (*LETM1*), Msh homeobox 1 (*MSX1*) and *WHSC1* (Dimmer et al., 2008; Simon and Bergemann, 2008; Zollino et al., 2003). Although most of the deletions are *de novo*, approximately 20% of the cases arise from unbalanced chromosome translocation t(4;8) (p16;23) inherited from either parent, probably as a result of gene duplication (Bergemann et al., 2005). *WHSC1* is considered the top candidate gene because it is consistently haploinsufficient (as part of a larger deletion) in all known cases of WHS (Bergemann et al., 2005; Van Buggenhout et al., 2004).

WHSC1 functions as a histone methyltransferase (HMT) to regulate gene expression in both embryonic and adult tissue (Brito et al., 2009; Martinez-Garcia et al., 2011). However, although the activity of WHSC1 is controversial, the consensus is that it catalyses methylation of lysine 36 residues on histone 3 (H3K36me), when presented with nucleosomes, the main components of chromatin (Li et al., 2009; Marango et al., 2008; Wagner and Carpenter, 2012). Like all other H3K36-specific HMTs identified thus far, WHSC1 contains the catalytic SET domain (Wagner and Carpenter, 2012). It also contains the chromatin-binding domain, proline-tryptophan-tryptophan-proline (PWWP), which interacts with H3K36me, a plant homeodomain (PHD) and a high-mobility group (HMG) DNA-binding domain (Wagner and Carpenter, 2012). The HMG domain of WHSC1 can interact with the DNA-binding domain of the androgen receptor (AR) and, in the presence of the ligand, enhances AR-mediated transcriptional activation, thereby implicating WHSC1 in the promotion of prostate carcinogenesis (Kang et al., 2009).

WHS is a contiguous gene syndrome in which the deletion size varies among affected individuals, with larger deletions resulting in more severe phenotypes. Prognosis thus depends on the diagnosis: most severe cases are stillborn; ∼35% die within 2 years, and those who survive into adulthood only make slow but steady progress in growth (Shannon et al., 2001; Zollino et al., 2003). The major features of the syndrome include a distinctive craniofacial appearance (broad, flat nasal bridge, prominent glabella, short philtrum, micrognathia and ocular hypertelorism), short stature due to growth retardation and global developmental delay, intellectual disability, and seizures. Speech problems, genitourinary abnormalities and other craniofacial manifestations such as proptosis, cleft palate, cleft lip and defective dentition are also common (Battaglia et al., 1999, 2001, 2008; Bergemann et al., 2005; Maas et al., 2008; Shannon et al., 2001; Tachdjian et al., 1992; Van Borsel et al., 2004; Verbrugge et al., 2009; Zollino et al., 2008). The deletion of *WHSC1* is associated with many characteristic WHS features, including the distinctive facial appearance (Bergemann et al., 2005; Van Buggenhout et al., 2004). *WHSC1* mouse mutant phenotypes resemble some WHS phenotypic features in human, including developmental delay, growth retardation, and heart, midline and craniofacial defects (Nimura et al., 2009). Whereas heterozygous mice are viable and show varying degrees of the WHS phenotype, homozygous mice show more severe phenotype and die shortly after birth (Nimura et al., 2009). Mouse knockout studies associate *LETM1* deletion with seizures and abnormal neuronal activity (Zollino et al., 2003, 2008), whereas dental and cleft abnormalities might be due to loss of *MSX1* function (Nieminen et al., 2003).

**TRANSLATIONAL IMPACT**

**Clinical issue**

Wolf-Hirschhorn syndrome (WHS) is a rare genetic disorder in humans that causes severe growth retardation, seizures and characteristic craniofacial defects. Affected individuals can also present with heart defects, cleft lip and/or palate, hearing impairment and eye anomalies. WHS is caused by the partial deletion of the short arm of chromosome 4, which harbours two overlapping critical regions (WHSCR-1 and WHSCR-2) consisting of multiple genes. Phenotypic variability and severity of the syndrome is determined by the extent of the deletion in these regions. The only gene common to both critical regions is *WHSC1*, which is also deleted in every known case of WHS, and thus is considered to be the best candidate causative gene for the syndrome. WHSC1 is a methyltransferase that regulates gene expression by modifying histone tails, specifically H3K36. It plays essential roles in cell cycle regulation, transcription and DNA repair via epigenetic modifications. The *WHSC1* mutant mouse is amongst the few animal models developed for WHS. However, the contribution of individual genes within the WHS critical regions to different phenotypes often cannot be established firmly and, in particular, the causes of sensorineural deafness in WHS have so far not been established.

**Results**

In this study, the authors used *WHSC1*-deficient mice, which display craniofacial defects similar to those seen in humans with WHS. Using this mouse model, the authors show that loss of WHSC1 function leads to characteristic sensory hair cell defects. Although overall hair cell numbers are normal, both inner and outer hair cells are disorganised with occasional extra rows at the base and none at the apex of the cochlea. In addition, the hair cell polarity is disturbed and their stereocilia bundles, which are required for sound perception, fail to develop the appropriate morphology. Finally, the authors provide evidence that hair cell innvervation is defective, which might contribute to sensorineural hearing loss.

**Implications and future directions**

These results reveal an important role for *WHSC1* in auditory hair cell development, particularly during cellular organisation and stereocilia morphogenesis, and in hair cell innervation. These alterations might be responsible for sensorineural hearing loss in human WHS. Furthermore, the results provide new insights into the epigenetic regulation of hair cell polarity and suggest that this activity is crucial for the arrangement of cochlear hair cells and their stereocilia. Because epigenetic modifications by WHSC1 are reversible, they are excellent targets for drug therapy in WHS.

The syndrome is also characterised by otological manifestations such as poorly formed ears (microtia), nystagmus, preauricular cysts or fistula (pits), epicanthal folds, low-set ears, otitis media, and hearing loss (Battaglia et al., 1999, 2001, 2008; Bergemann et al., 2005; Chen et al., 2011; Maas et al., 2008; Shannon et al., 2001; Tachdjian et al., 1992; Van Borsel et al., 2004; Verbrugge et al., 2009; Zollino et al., 2008). Yet, the causative gene (or genes) for hearing loss in WHS has not been identified. Deletion of *FGFR3* might contribute to hearing loss: *FGFR3^−/−^* mice are deaf owing to disorganisation of cochlear supporting cells and possibly innervation of the outer hair cells (Colvin et al., 1996; Hayashi et al., 2007; Mansour et al., 2009, 2013; Puligilla et al., 2007). Although hearing loss in individuals with WHS is mostly conductive, and often secondary to chronic otitis media (Battaglia et al., 1999, 2008; Lesperance et al., 1998; Ulualp et al., 2004), clinical studies also identified a number of cases with sensorineural hearing loss, both with and without conductive hearing loss (Battaglia and Carey, 1999; Lesperance et al., 1995, 1998; Simon and Bergemann, 2008). However, the molecular and cellular mechanisms responsible for sensorineural defects are not known.

In this study, we examine the inner ears of *WHSC1*-deficient mice and describe for the first time defects in auditory hair cells of the cochlea associated with loss of WHSC1. Our observations provide new insights into the role of histone-modifying enzymes in cochlear development and into the pathophysiology of sensorineural hearing loss in individuals with WHS.

## RESULTS

### Craniofacial manifestation of WHS clinical features in *WHSC1^−/−^* mice

**Fig. 1.**
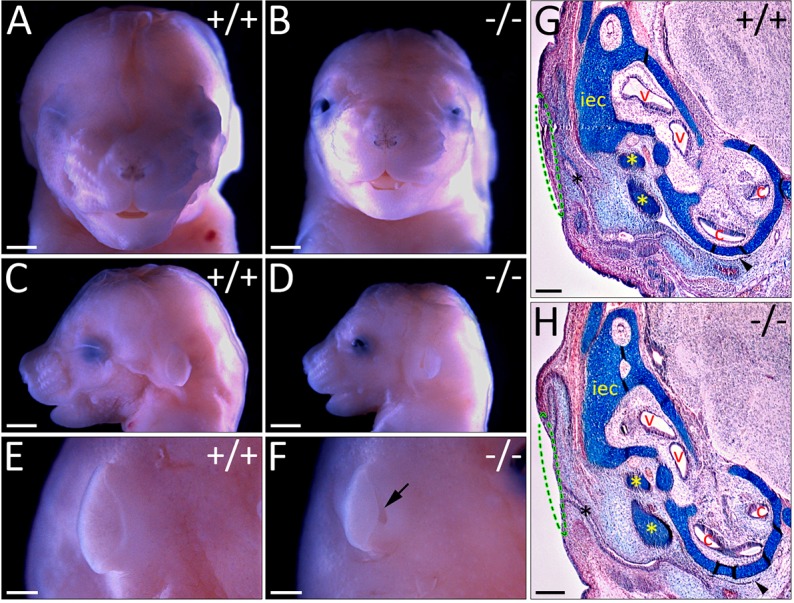
**Craniofacial phenotype of *WHSC1* mutant mice.** E16.5 wild-type and mutant heads (A-F) and transverse sections (G,H) stained for Picro-Sirius red trichrome. *WHSC1^−/−^* mice have the characteristic broad, flat nasal bridge, prominent glabella, short philtrum and micrognathia observed in individuals with WHS, as well as microtia and hypertelorism. The middle and inner ear morphology are normal. Scale bars: 500 μm (A-F); 200 μm (G,H). Arrow, pit in the outer ear; arrowhead, eustachian tube; black asterisk, ear canal; yellow asterisk, middle ear bones; iec, inner ear capsule; v, vestibule; c, cochlea.

The characteristic craniofacial dysmorphism of individuals with WHS is associated with haploinsufficiency of the *WHSC1* gene (Bergemann et al., 2005; Van Buggenhout et al., 2004) and is replicated in *WHSC1* mutant mice (Nimura et al., 2009). Here, we confirm the published phenotypes. *WHSC1^−/−^* mutants are developmentally delayed and are smaller in size compared to control or heterozygous littermates (Nimura et al., 2009). Close examination of the craniofacial structures reveals a decrease in the size of *WHSC1^−/−^* mutant heads by 22% (1230±9 μm, *n*=3 vs 1577±4 μm, *n*=3; *P*=4×10^7^, Fig. 1A-D) and a temporal protrusion of the skull (supplementary material Fig. S1B,C,E,F, arrowheads) that also characterises individuals with WHS. Furthermore, *WHSC1^−/−^* mice have open eyelids, with widely spaced protruding eyes compared to *WHSC1^+/+^* or *WHSC1^+/−^* littermates (Fig. 1A,B; not shown). The protruding-eye phenotype is also observed in some individuals with WHS with hypertelorism (Bergemann et al., 2005; Van Buggenhout et al., 2004). About 15% of individuals with WHS exhibit sensorineural hearing loss (Toriello and Smith, 2013), although the cause remains unknown. To determine whether sensorineural hearing loss is associated with *WHSC1* deletion, we analysed inner ear development in *WHSC1* mutant mice. These mutants do not survive to postnatal day 10 (P10) and the number of viable *WHSC1^−/−^* embryos are greatly reduced from late embryonic stages (Nimura et al., 2009). We therefore focused on E16.5 and E18.5, when the inner ear is fully formed and sensory hair cells are at different stages of differentiation and maturation (Chen and Segil, 1999). *WHSC1* mutants form pits in the outer ear (Fig. 1E,F, arrow), similar to phenotypes observed in individuals with WHS presenting with preauricular cysts/pits and/or epicanthal folds (Bergemann et al., 2005; Van Buggenhout et al., 2004). Sections reveal the loss or underdevelopment (i.e. microtia) of the pinnae that results in pit formation (Fig. 1G,H, green dashed line; supplementary material Fig. S2). The middle ear bones and the inner ear are largely normal, as is the eustachian tube and ear canal, even though they appear slightly constricted (Fig. 1G,H; supplementary material Fig. S2, dotted lines). Collectively, our observations confirm that *WHSC1* mutants phenocopy WHS characteristics attributed to the deletion of *WHSC1*, including craniofacial abnormalities, and external ear and eye anomalies.

### *WHSC1* is not required for the formation of hair cells

**Fig. 2.**
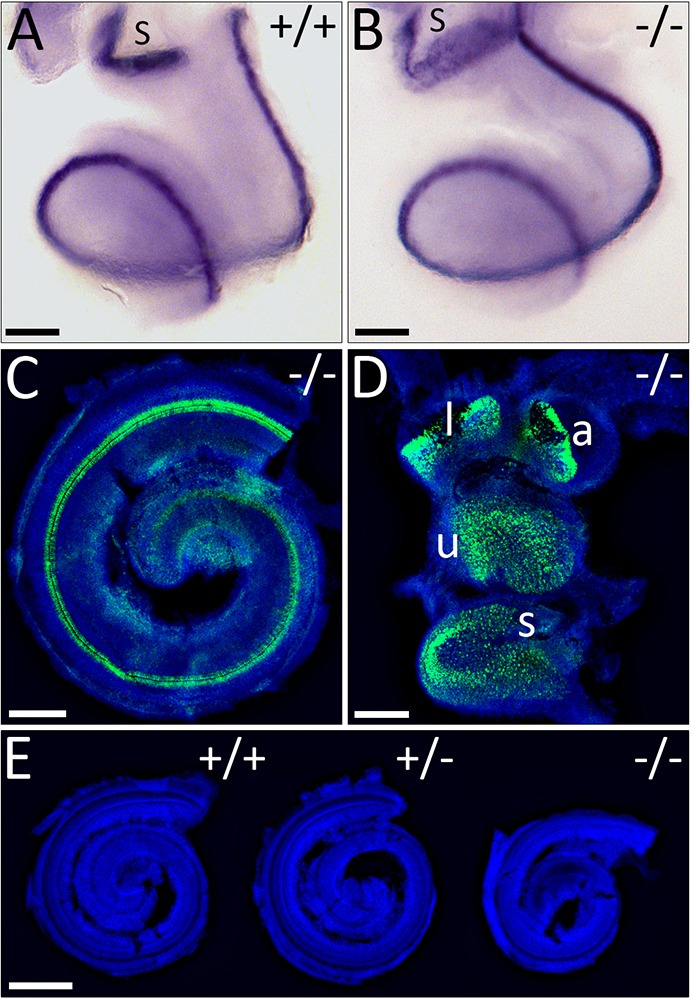
**Development of cochlear and vestibular hair cells.** (A,B) E16.5 wild-type and mutant cochlea revealing *ATOH1* expression. (C,D) Immunolabelling of mutant cochlea and vestibule for MYO7A (green) counterstained with Hoechst to label nuclei (blue). Auditory and vestibular hair cell development is normal in *WHSC1^−/−^* mice. (E) E18.5 cochleae stained with Hoechst. Scale bars: 200 μm (A-D); 500 μm (E). l, lateral crista; a, anterior crista; u, utricle; s, saccule.

Although *WHSC1* mutant inner ears are smaller, their gross morphology is normal. We next sought to determine whether the cellular anatomy of the cochlear and vestibular structures is affected in *WHSC1* mutants. The transcription factor *ATOH1* is expressed in cells committed to the hair cell lineage and functions upstream of the differentiation marker *MYO7A* (Bermingham et al., 1999; Chen and Segil, 1999). *ATOH1* is expressed normally in vestibular (not shown) and in auditory hair cells in both *WHSC1^−/−^* and *WHSC1^+/−^* inner ears (Fig. 2A,B; not shown). The size of the cochlea was slightly, but not significantly, shorter in heterozygous animals (3264±41 μm vs 3276±65 μm; *n*=3, *P*=0.8), but significantly decreased by 22% (2541±113 μm vs 3276±65 μm; *n*=3, *P*=0.002) in homozygous mutants (Fig. 2E), consistent with a global reduction in the size of the *WHSC1*^−/−^ mutants. However, the number of cochlear turns was unaffected (Fig. 2A,B) and the expression of MYO7A was initiated normally (Fig. 2C,D), suggesting that *WHSC1* is not required for cochlear duct elongation or the formation and differentiation of hair cells.

### Loss of WHSC1 function disrupts cochlear hair cell organisation

**Fig. 3.**
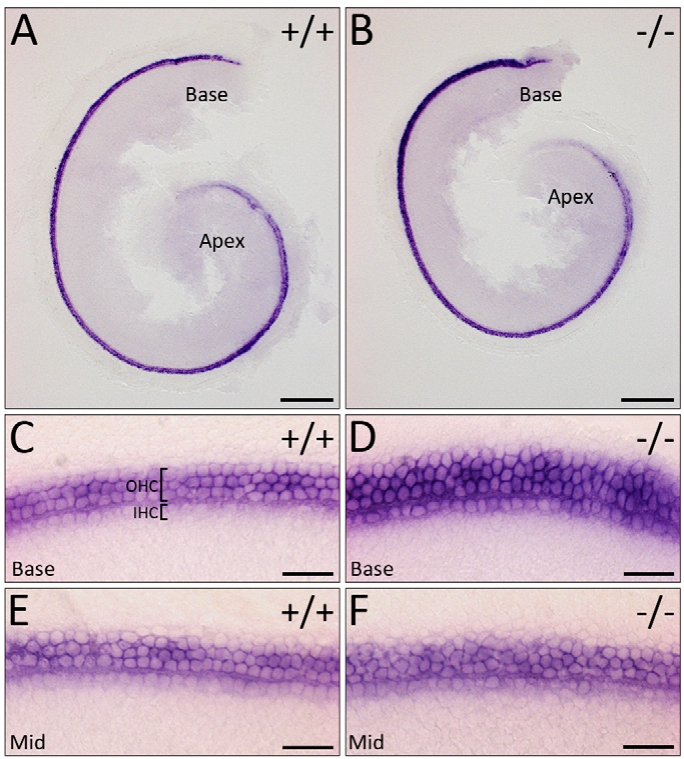
**Cochlear hair cells fail to arrange into organised rows.** (A,B) Low-power images of E16.5 cochlea showing *ATOH1* expression. (C-F) High-power images of E16.5 cochlear hair cells showing normal expression of *ATOH1* in *WHSC1^−/−^* mice but failure to arrange into organised rows. Scale bars: 200 μm (A,B); 25 μm (C-F). OHC, outer hair cell; IHC, inner hair cell.

**Fig. 4.**
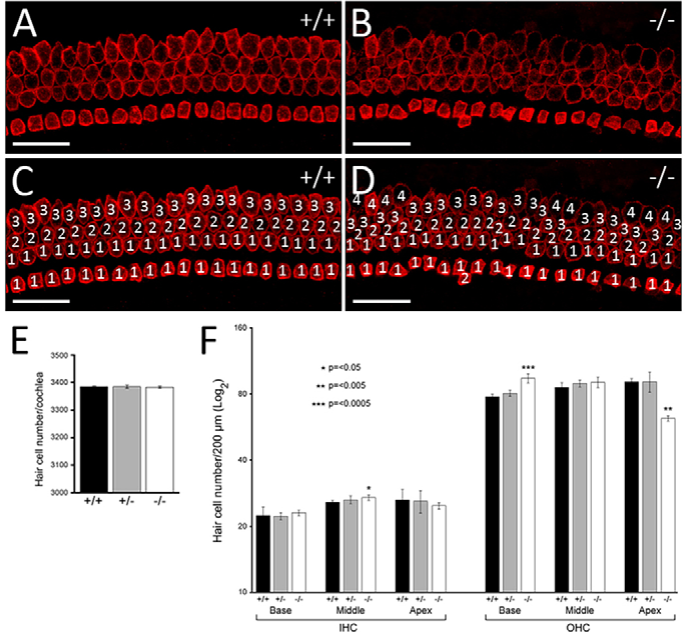
**Extra hair cells in the basal and middle regions of the cochlea.** (A-D) E18.5 wild-type and mutant organ of Corti immunostained for MYO7A (shown for basal turn of the cochlea). The extra rows of hair cells result from the disorganised arrangement of the hair cells. (E) Quantification of the total number of hair cells in cochleae of *WHSC1^+/+^*, *WHSC1^+/−^* and *WHSC1^−/−^* littermates showed no significant difference (*n*=3, *P*=0.4). (F) Quantification of the total number of inner hair cells (IHCs) and outer hair cells (OHCs) in a 200-μm region of base, middle and apical turns of the cochleae in heterozygous and homozygous mutants and control littermates (*n*=5). Note the significant increase in the number of IHCs in the middle (*P≤0.05) and OHCs in the base (***P≤0.0005), with a concomitant decrease of OHCs in the apex (**P≤0.005) of *WHSC1^−/−^* cochlea. Error bars are s.e.m. Scale bars: 25 μm.

Although *ATOH1* is expressed normally and hair cells differentiate along the length of the cochlea, we noticed a stronger and wider expression domain of *ATOH1* at the basal and middle turns of the cochlea in mutants (Fig. 3A,B), prompting us to investigate the hair cell arrangement in more detail. Normally, cochlear hair cells are arranged in organised rows, with one row of inner hair cells (IHCs) and three rows of outer hair cells (OHCs) (Fig. 3C). At E16.5, this organisation is already observed in the basal and mid-basal turns of the cochlea, whereas mid-apical and apical (except the apical tip) hair cells become organised by E18.5, because these are the last to mature, albeit the first to exit the cell cycle (Chen and Segil, 1999; Matei et al., 2005). Instead of four discernible rows, the mutant cochlea showed multiple (and somewhat ambiguous) hair cell rows (Fig. 3C-F; *n*=4/5). Although it is possible that this arrangement is due to a developmental delay, this is unlikely because we observe the same phenotype at E18.5 (Fig. 4A-D; *n*=14/14): there are extra IHC and/or OHC rows, ranging from the normal 4 rows to an abnormal 6 rows in the basal and middle regions of *WHSC1^−/−^* cochlea (Fig. 4C,D; *n*=14/14). A small proportion of heterozygous *WHSC1^+/−^* cochlea (20%) show the same hair cell phenotype (*n*=8/40; supplementary material Fig. S3G,I, arrowheads). In the mutant cochlea, the total number of hair cells did not significantly differ compared to wild-type controls (3382±5 vs 3385±2; *n*=3) or heterozygous littermates (3385±6 vs 3385±2; *n*=3; Fig. 4E), suggesting that it is the failure of hair cell arrangement along the length of the cochlear duct that ultimately results in their inappropriate accumulation in some regions. In support of this notion, the last set of differentiating hair cells in the apical turn appeared more sparsely distributed, probably due to reduced numbers in this region (Fig. 3A,B). This observation was confirmed at E18.5, where MYO7A^+^ hair cells were strongly reduced in the apical tip of the mutant cochlea when compared to controls (supplementary material Fig. S4C,D). In particular, the number of OHCs were significantly reduced in the mutant apex (62±2 vs 91±3; *n*=5, *P*=0.001), with a corresponding increase in the base (94±4 μm vs 78±2 μm; *n*=5, *P*≤0.001; Fig. 4F). The number of IHCs was less affected, with a slight but significant increase in the middle of the cochlea (27±0.7 vs 26±0.6; *n*=5, *P*=0.03; Fig. 4F). *WHSC1^+/−^* cochleae do not show any statistically significant changes because the phenotype is more subtle, but there are visibly more OHCs in the base (80±3 vs 78±2; *n*=5, *P*=0.2) and middle (89±3 vs 85±5; *n*=5, *P*=0.3) turns of the cochlea (Fig. 4F, supplementary material Fig. S3G,I, arrowheads). Thus, WHSC1 function is required as hair cells begin to align and arrange themselves into distinct rows along the length of the cochlea.

### *WHSC1* mutants show abnormal hair cell shape and size but normal pillar cells

**Fig. 5.**
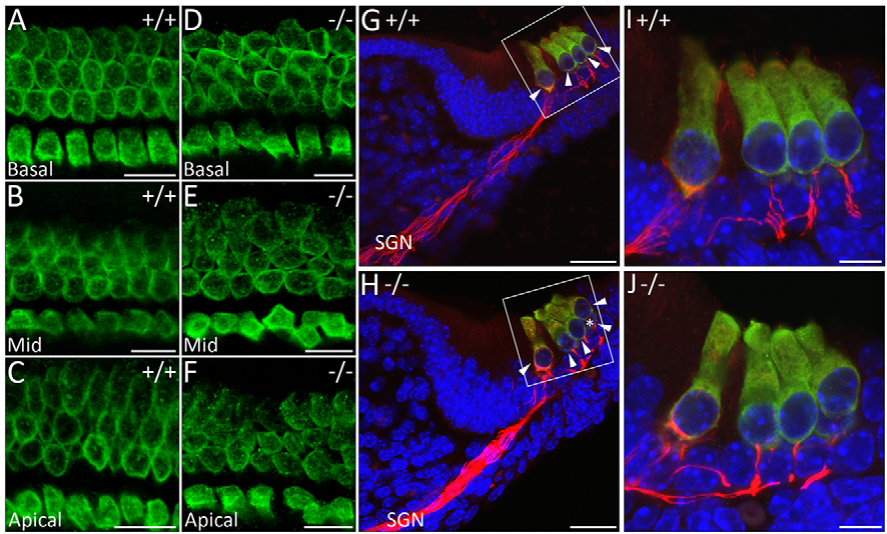
**Auditory hair cells are disorganised in *WHSC1*^−/−^ mice.** (A-F) E18.5 wild-type and mutant organ of Corti immunostained for MYO7A (green). Differences in cell shape and size affect the arrangement of hair cells. (G-J) MYO7A and neurofilament (red) staining in sections show more spiral ganglia neuron (SGN) fibres towards outer hair cells in *WHSC1*^−/−^ mice; nuclei stained with Hoechst (blue). Scale bars: 10 μm (A-F,I,J); 30 μm (G,H). (I,J) Higher magnifications of the white boxed regions in G and H, respectively. Arrowheads, hair cells; asterisk, hair cell squeezed in between other hair cells. Mid, middle region of the cochlea.

To investigate the hair cell phenotype further, we analysed E18.5 cochlea immunostained for MYO7A by confocal microscopy. We found a striking difference in size and shape between mutant and wild-type cochlear hair cells. Whereas all hair cells appeared similar in size in *WHSC1^+/+^* cochlea, their size varied from normal to small in *WHSC1^−/−^* mice (Fig. 4A,B), and to a lesser extent in *WHSC1^+/−^* mice (supplementary material Fig. S3G). These differences seem to affect the intercellular space: in mutant mice some hair cells made contact with many other cells (without any intercellular gaps), whereas other cells (especially IHCs) are more spaced out, not establishing any contacts at all (Fig. 5A-F). This hair cell disorganisation was also observed in sections (Fig. 5G-J, arrowheads), where extra cells seem to be squeezed between existing cells (in this case OHCs, Fig. 5H,J, asterisk). The inner pillar cells were present and seemed to be normal (supplementary material Fig. S3), although one inner pillar cell was missing in *WHSC1^+/−^* cochlea (supplementary material Fig. S3I,J, arrow; *n*=1/13).

### Innervation is disrupted in *WHSC1^+/−^* and *WHSC1^−/−^* cochleae

**Fig. 6.**
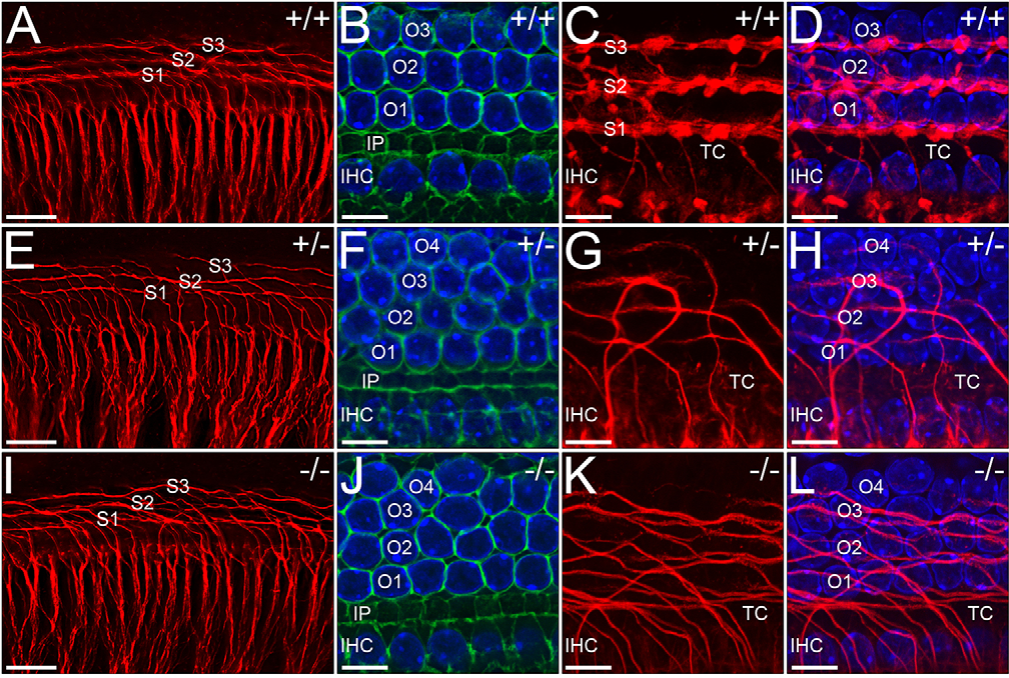
**Organisation of spiral fibres is disrupted in *WHSC1* mutant cochleae.** Basal region of E18.5 wild-type and mutant organ of Corti immunostained for neurofilament (red), phalloidin (green) and Hoechst (blue). The innervations pattern appears normal as spiral ganglia fibres project towards their hair cell targets (A,E,I). However, whereas fibres crossing the tunnel of Corti (TC) turn basally and fasciculate to form three distinct rows in wild type (C,D), they fail to fasciculate in heterozygous and homozygous mutants (G,H,K,L). Note the orderly arrangement of similarly shaped and sized hair cells in wild-type (B) versus disorganised arrangement in mutant (F,J) cochleae. Scale bars: 30 μm (A,E,I); 10 μm (B-D,F-H,J-L). TC, tunnel of Corti; S1-S3, spiral fibre rows; O1-O4, outer hair cell rows; IHC, inner hair cells.

As sensorineural hearing loss can also be caused by abnormalities in the bipolar spiral ganglia neurons that innervate hair cells, we performed immunolabelling for neurofilament (NF) to detect neuronal fibres. Spiral ganglion projections to IHCs appear to be normal but more fibres seem to target the OHCs (Figs 5G-J and 6). In *WHSC1^+/+^* cochleae, the majority of fibres terminate on the IHCs, while some cross the tunnel of Corti, turn towards the base and form three rows of spiral fibres (Fig. 6A-D). This arrangement of fibres is disrupted in both *WHSC1^+/−^* (Fig. 6E-H; *n*=3/25) and *WHSC1^−/−^* (Fig. 6I-L; *n*=7/11) cochleae. Together these findings suggest that *WHSC1* function is required for the organisation of spiral fibres, in addition to hair cell arrangement.

### Stereocilia formation is severely perturbed in *WHSC1* mutant cochlea

**Fig. 7.**
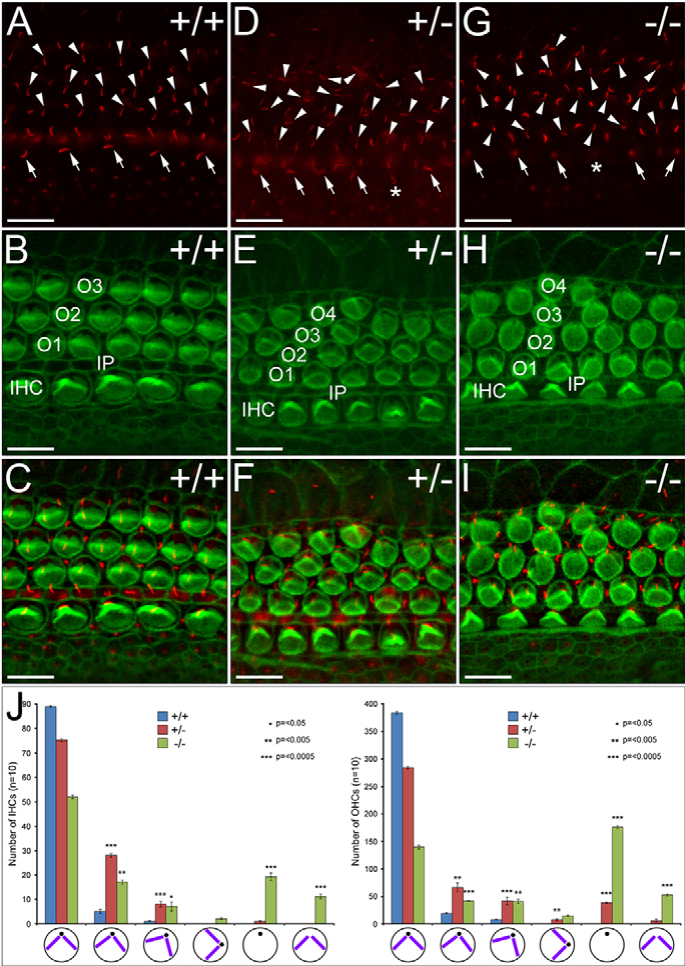
**Stereocilia formation is perturbed in *WHSC1^−/−^* hair cells.** (A-I) Basal region of E18.5 wild-type and mutant organ of Corti labelled with acetylated α-tubulin to visualise kinocilia (red) and phalloidin to reveal stereocilia (green). Kinocilia and stereocilia formation is abnormal in the mutant cochlea. (J) Quantification and schematic representation of the hair bundle defects in wild-type (*n*=10), heterozygous (*n*=10) and homozygous (*n*=10) animals. In mutants, the kinocilia (black dots) and stereocilia (purple) are either absent or have largely abnormal orientation. Those that do have a kinocilia can either have no stereociliary hair bundles or these bundles are localised around the cell perimeter. Scale bars: 10 μm. Asterisk: (D) central position of kinocilia rather than peripheral; (G) no kinocilium. Arrowheads, outer hair cell kinocilia; arrows, inner hair cell kinocilia.

Hair cell stereocilia are crucial for the function of these cells as mechanosensory cells and to transmit sound information to the auditory nuclei in the brain stem (Yoshida and Liberman, 1999). Stereocilia develop on the apical surface of each hair cell and they can be visualised by Phalloidin to label F-actin polymers. Before morphogenesis of the hair bundle stereocilia, a specialised primary cilium, the kinocilium, visualised by acetylated α-tubulin, forms at the apical surface of each hair cell. As the kinocilium moves towards the cell periphery, actin stereocilia begin to form around it into a distinct morphology (inverted ‘V’ in OHCs and ‘C’ in IHCs). This arrangement of the hair bundle stereocilia is a manifestation of planar cell polarity (PCP; Ezan and Montcouquiol, 2013; Goodrich and Strutt, 2011; Sienknecht et al., 2014). At E18.5, hair cells in the base of the cochlea already show appropriate stereocilia morphology in control animals: they are aligned and orientated in the same direction (Fig. 7A-C; supplementary material Fig. S3A). Although kinocilia and stereocilia are present in *WHSC1^+/−^* and *WHSC1^−/−^* mutants, they exhibit abnormal shape, length and orientation (Fig. 7D-J; supplementary material Fig. S3G,M). Frequently, stereociliary hair bundles were localised around the cell periphery. In homozygous *WHSC1^−/−^* mice, a substantial proportion of OHCs and IHCs lacked kinocilia or stereocilia (Fig. 7D-J; supplementary material Fig. S3G,M; asterisk). Taken together, our data suggest that *WHSC1* is required during hair cell differentiation as the cells begin to organise themselves into distinct rows and form stereocilia, orientated in one direction.

## DISCUSSION

Individuals with WHS show varying phenotypes, including sensorineural and conductive hearing loss in approximately 40% of patients (Battaglia et al., 2008). To elucidate the possible cause of sensory defects, we analysed the inner ear of the WHSC1 mouse model for WHS. Our results reveal an important role for *WHSC1* in auditory hair cell development, particularly during cellular organisation and stereocilia morphogenesis, and in hair cell innervation. Furthermore, the results provide new insights into the epigenetic regulation of hair cell polarity. Individuals with WHS commonly present with otological manifestations such as preauricular and/or auricular abnormalities, and, of them, almost half have chronic otitis media with effusion (Lesperance et al., 1998). In addition, some patients also display sensorineural hearing loss, and a smaller percentage still might have missing OHCs in areas within the basal region of the organ of Corti, but only in one ear (Ulualp et al., 2004). In the organ of Corti of *WHSC1^−/−^* mice, IHCs and OHCs are missing within the apical turn in both ears. We provide evidence that this is not due to the lack of hair cell differentiation. Rather, we find that the hair cells fail to arrange appropriately along the length of the cochlea, resulting in their accumulation in the basal and medial turns. We also show that hair cell innervation is disrupted, which might contribute to sensorineural hearing loss. Our data identify haploinsufficiency of *WHSC1* as a potential cause of sensorineural hearing loss in individuals with WHS.

### WHSC1 and PCP signalling

Disorganisation of hair cells is a hallmark of defects in planar cell polarity (Ezan and Montcouquiol, 2013; Goodrich and Strutt, 2011; Sienknecht et al., 2014). Hair cells display both an extrinsic and intrinsic level of polarity, i.e. they are arranged in an organised pattern along the cochlea and stereocilia form at their apical surface (Ezan and Montcouquiol, 2013; Sienknecht et al., 2014; Tarchini et al., 2013). During its development, the cochlear epithelium elongates via convergent-extension movements, a mechanism controlled by core PCP signalling mediated through the RAC-JNK pathway. Defects in the core PCP components such as *VANGL* result in a shortened cochlear duct and the failure of hair cells to organise appropriately, including the orientation (but not the formation) of stereociliary bundles (Ezan and Montcouquiol, 2013; Sienknecht et al., 2014; Torban et al., 2008). Mutations in a parallel pathway, the FAT signalling pathway, also result in hair cell defects, and *VANGL2* and *FAT4* genetically interact (Ezan and Montcouquiol, 2013; Saburi et al., 2008; Sienknecht et al., 2014). We observed the *WHSC1* mutant phenotype at the same time as when hair cells respond to these extrinsic tissue polarity cues to organise themselves. Moreover, hair cells interpret these cues to drive cell-intrinsic polarity by positioning the basal body appropriately, which ultimately leads to an organised stereociliary hair bundle. This step is independent of core PCP genes, but involves RAC-PAK signalling, with LIS1 regulating localised RAC-PAK signalling, and maintaining stereocilia bundles during both fetal and postnatal development (Sipe et al., 2013). Conditional inactivation of *LIS1* hair cells (*LIS1^cKO^*) affects their organisation into distinctive rows, as well as cell shape and size and stereocilia formation, although the cochlea length is normal (Sipe et al., 2013). Thus, the *WHSC1^−/−^* phenotype described here is remarkably similar to *LIS1*^*cKO*^, but does not correlate with defects observed in core PCP mutants. Although hair cell bundles are determined by positioning of the basal body, the organised rows of hair cells are regulated by a family of cell adhesion molecules called NECTINS (Togashi et al., 2011). NECTIN1, -2 and -3 are localised at the cell-cell contacts between hair cells, and the cellular organisation defects in *LIS1^cKO^* organ of Corti are likely to be caused by impaired NECTIN-mediated cell adhesion (Sipe et al., 2013; Fukuda et al., 2014). Loss of *MYO7A* in mice also results in stereocilia defects (Self et al., 1998); however, in both *LIS1* and *WHSC1* mutants, MYO7A is expressed normally. Thus, the strong correlation of *WHSC1* and *LIS1* mutant phenotypes suggests that both might function in the same pathway. Future experiments will need to address the epistatic relationship between *LIS1* and *WHSC1*, and the question of whether WHSC1 transcriptionally regulates *LIS1* through histone modification.

### Contribution of WHSC1 and FGFR3 deletion to deafness in individuals with WHS

Amongst the genes found in the WHSCR-2 is *FGFR3*, which, when deleted in mice, results in profoundly deaf animals (Colvin et al., 1996; Puligilla et al., 2007). However, although hair cells in *FGFR3^−/−^* mice are normal, a subset of supporting cells, which are crucial for hair cell function, are defective. Specifically, inner and outer pillar cells, although present, fail to differentiate (Colvin et al., 1996; Hayashi et al., 2007; Puligilla et al., 2007). In addition, the sensory epithelium is wider, with an extra row of OHCs in the apex of the cochlea, accompanied by an increase in the number of Deiters' cells (Hayashi et al., 2007). Inner pillar cells show limited p75^NTR^ expression (Puligilla et al., 2007), but it is unclear whether these cells are completely absent or transformed into another cell type. In the *FGFR3* mouse model for Muenke syndrome (*FGFR3^P244R/+^*), Deiters' cells transform into pillar cells (Mansour et al., 2009) and these two types of supporting cells can reversibly switch fates in an FGF-dependent manner (Mansour et al., 2013). In addition to supporting-cell defects, the outer hair cells and their innervation by spiral ganglia fibres are also disrupted (Puligilla et al., 2007). Thus, FGF signalling via *FGFR3* regulates the fate decision between two populations of supporting cells (Colvin et al., 1996; Hayashi et al., 2007; Mansour et al., 2009, 2013; Puligilla et al., 2007), influencing hair cell function indirectly. Therefore, *FGFR3*, like *WHSC1*, might contribute to sensorineural hearing loss in individuals with WHS (Battaglia and Carey, 1999). However, the process by which they cause hearing impairment differs considerably, although, remarkably, in both *FGFR3* and *WHSC1* mutants, the spiral fibres fail to organise into distinct rows.

Individuals with WHS present with variable phenotypes. It is likely that deletion of both *WHSC1* and *FGFR3* contributes to sensorineural hearing loss in severe cases with large deletions in the critical region. However, hearing defects in individuals with smaller deletions are likely to be associated with *WHSC1* haploinsufficiency alone because this is observed in every known case of WHS, whereas *FGFR3* deletion is not. The incidence of sensorineural hearing loss in WHS is approximately 15% out of 40% of patients that present with hearing loss (Battaglia et al., 2008; Toriello and Smith, 2013). Interestingly, our results reveal that only 20% of the heterozygous *WHSC1^+/−^* cochlea show a hair cell phenotype, suggesting that these mice model the human syndrome quite accurately. Furthermore, it suggests that more patients with conductive hearing loss might also exhibit sensorineural hearing loss than currently identified. As with all syndromes with intellectual disabilities, prognosis is often better if hearing loss can be diagnosed as early as possible. The identification of WHSC1 as an important histone modifier whose activity is necessary for stereocilia development opens a new route to our understanding of cochlea biology and hearing loss.

### Complexity of WHSC1 function

The variability of the defects observed in individuals with WHS and in *WHSC1*^−/−^ mice might be due to the interaction of WHSC1 with different proteins. During heart development, WHSC1 interacts with and negatively modulates the transcriptional activity of NKX2.5, an important heart determinant (Nimura et al., 2009). WHSC1 also interacts with β-catenin and transcriptionally regulates the cell cycle regulator CCND1 through H3K36 trimethylation (Toyokawa et al., 2011). More recently, BRD4 and P-TEFb were identified as WHSC1 interacting partners, and together facilitate transcriptional elongation (Sarai et al., 2013). Furthermore, WHSC1 and HIRA, a histone H3.3-specific chaperone, interact to prolong H3.3 incorporation into activated genes (Sarai et al., 2013). In addition to histone methylation, WHSC1 promotes recruitment of NF-κB and p300 complexes to the promoters of NF-κB target genes, resulting in increased transcription of target genes, including of *WHSC1* itself (Yang et al., 2012). Finally, WHSC1 is phosphorylated in response to DNA double-strand breaks (Matsuoka et al., 2007; Pei et al., 2011), which mediates its recruitment to sites of DNA damage where it dimethylates H4K20 to recruit 53BP1 and promote DNA repair (Hajdu et al., 2011; Pei et al., 2011). This identifies WHSC1 as a component of the DNA damage response (DDR) machinery, and it has been proposed that the features of WHS attributed to *WHSC1* function arise as a result of a defective DDR pathway (Hajdu et al., 2011). Whether this holds true for the cochlea remains to be determined because the specific interacting protein for WHSC1 in the organ of Corti has not been identified. However, because of the specific defects in *WHSC1^−/−^* inner ears, potential candidates are components of the PCP machinery.

### Conclusion

In summary, WHSC1 mutant mice provide a good model for understanding the causes of sensorineural hearing loss in individuals with WHS. We show that loss of WHSC1 function leads to the failure of hair cell organisation and of stereocilia formation in the cochlea, as well as innervation defects. Importantly, about 20% of heterozygous mice show a similar phenotype, matching closely with the incidence of sensorineural hearing loss observed in WHS-affected individuals. This is particularly exciting because epigenetic modifications such as histone methylation are reversible and therefore good drug targets.

## MATERIALS AND METHODS

### *WHSC1* mice

The breeding and generation of *WHSC1* mutant mice are described in Nimura et al. (2009). All animal work was performed in accordance with UK Home Office regulations.

### Histology and skeletal preparation

Embryos for histological analysis were decalcified in 10% EDTA/0.1 M Tris-HCl (pH 8.0) for 2 weeks at 4°C. This was followed by dehydration in an ascending ethanol series and embryos were processed for paraffin-wax sectioning (8 μm). Picro-Sirius red and alcian blue trichrome staining (Puchtler et al., 1973) was performed on the sections. Skeletal preparation was performed as described in Rice et al. (2003).

### *In-situ* hybridisation and immunohistochemistry

The inner ear was fixed in 4% para-formaldehyde (PFA) in phosphate-buffered saline (PBS) overnight at 4°C, then the cochlea and vestibule were dissected in DEPC-treated PBS. *In-situ* hybridisation was performed using Dig-labelled *ATOH1* riboprobe (gift from M. A. Basson, Craniofacial Development and Stem Cell Biology, King's College London, London, UK). Antibodies used were: rabbit MYO7A (1:1000, Proteus), rabbit acetylated α-tubulin (1:500, Abnova), rabbit p75^NTR^ (1:500, Abnova), detected with anti-rabbit Alexa Fluor 488 or 568 (1:1000, Invitrogen), mouse NF-M (1:100, Invitrogen), detected with anti-mouse Alexa Fluor 488 or 568 (1:1000, Invitrogen) and Alexa Fluor 488 or 568 Phalloidin (1:1000, Invitrogen).

### Microscopy and imaging

Images were captured using an SMZ1500 stereo microscope (Nikon), an Axiovert 200 M (Zeiss) compound microscope and a TCS SP5 confocal (Leica) microscope. Figure panels were assembled in Photoshop CS (Adobe).

### Cell counts and measurements

The total number of hair cells was counted on photographs of immunostained cochleae. For counts by cochlear region, the cochlear length was divided into equal basal, middle and apical portions, and the total number of hair cells was counted in a 200-µm field. Cochlear length was determined using Fiji (ImageJ) and drawing a line from the base of the cochlea to the apex. Statistical comparisons were made using unpaired, two-tailed Student's *t*-tests.

## Supplementary Material

Supplementary Material
