# Supplementary material for: Auditory hair cell defects as potential cause for sensorineural deafness in Wolf-Hirschhorn syndrome
Source: Dis Model Mech. 2015 Sep 1;8(9):1027–35. doi: 10.1242/dmm.019547 (PMC4582100; doi:10.1242/dmm.019547)
Supplement: Supplementary Material [file supp_8_9_1027__index.html]

Supplementary Material 

# Auditory hair cell defects as potential cause for sensorineural deafness in Wolf-Hirschhorn syndrome

## DMM019547 Supplementary Material

- Supplementary Material
