# Supplementary material for: Auditory hair cell defects as potential cause for sensorineural deafness in Wolf-Hirschhorn syndrome
Source: Dis Model Mech. 2015 Sep 1;8(9):1027–35. doi: 10.1242/dmm.019547 (PMC4582100; doi:10.1242/dmm.019547)
Supplement: Supplementary Material [file supp_019547_DMM019547supp.pdf]

Supplementary Figures

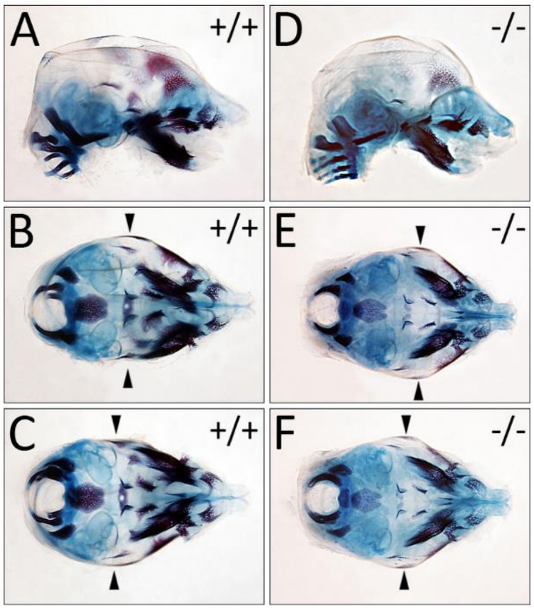

Figure S1: Skeletal preparation of *WHSC1* heads

Alcian blue and alizarin red staining of E16.5 wildtype and mutant heads. **A, D:** Lateral, **B, E:** dorsal and **C, F:** ventral views. Note the temporal protrusion of the skull (arrowheads in E, F compared with B, C). The inner ear cartilage and tympanic ring develop as normal. The developmental delay in ossification has been shown previously (Nimura et al., 2009).

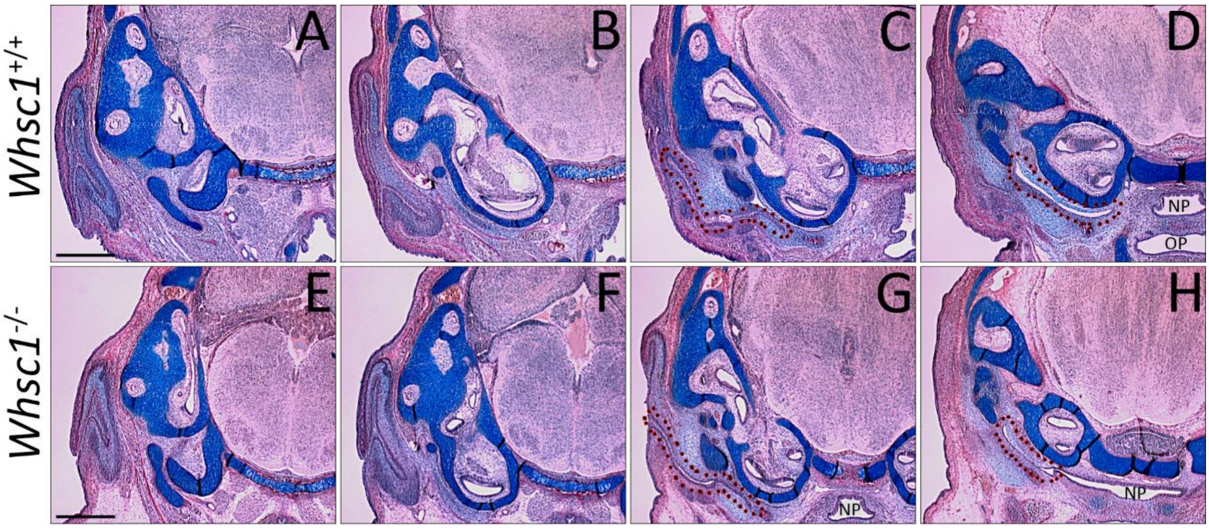

Figure S2: Craniofacial phenotype of *WHSC1* mutant mice

A-D: E16.5 wildtype and **E-H:** mutant transverse sections stained for Picro-Sirius red. The middle and inner ear are morphologically normal. Scale bars: 500µm. Dotted lines (C, G) = ear canal, dotted lines (D, H) = eustachian tube, NP = nasopharynx, OP = oropharynx.

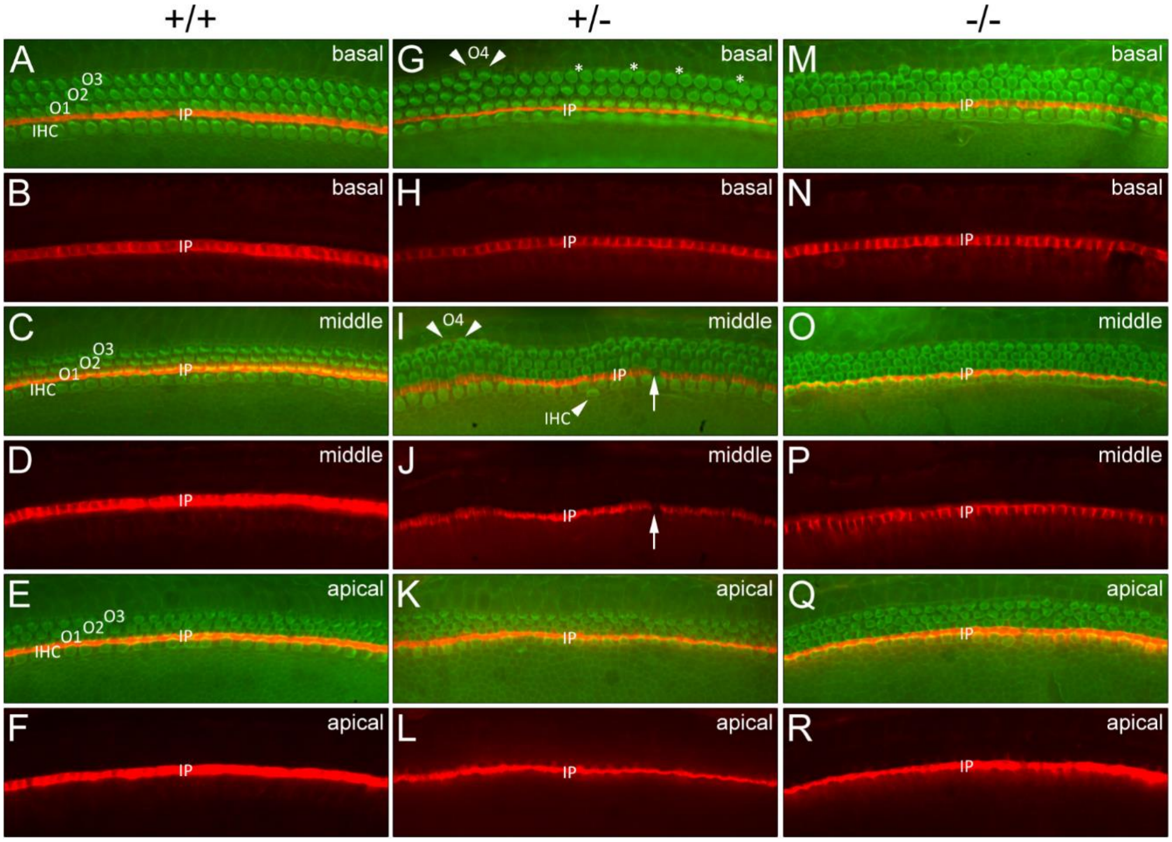

Figure S3: Defective hair cells but normal pillar cells in *WHSC1* mutant cochleae

Hair cells and their stereocilia are revealed with phalloidin (green). Inner pillar cells are present and normal, as revealed by $p75^{NTR}$ staining (red). Hair cells are arranged into four organised rows, with stereocilia orientated in one direction in *WHSC1*$^{+/+}$ (A-F), but not in *WHSC1*$^{+/-}$ (G-L) and *WHSC1*$^{-/-}$ (M-R) cochleae. IP = inner pillar cell, IHC = inner hair cell, O1-4 = outer hair cell rows, arrowheads = extra hair cells, asterisk = absent or reduced stereocilia formation, arrow = missing inner pillar cell. Scale bars: 30µm.

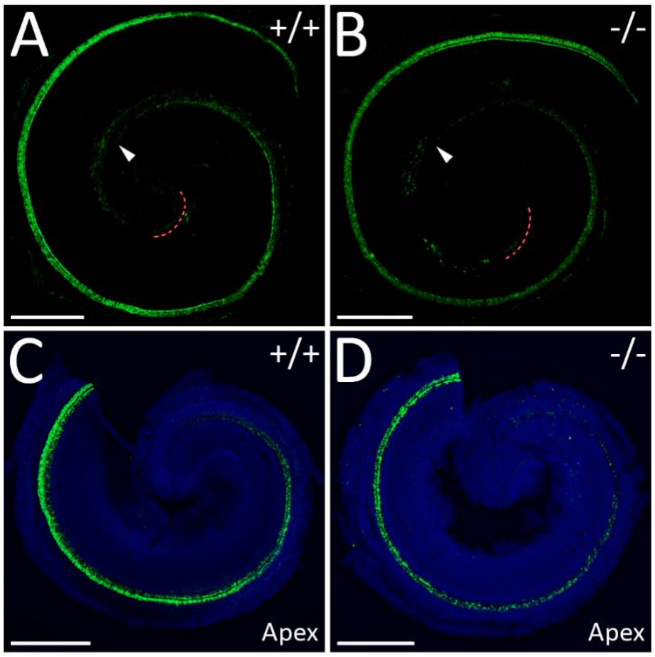

Figure S4: Reduced number of hair cells in the apex of the cochlea

A, B: E18.5 whole cochlea immunostained for MYO7A (green). **C, D:** apical region of the cochlea stained with MYO7A (green) and Hoechst (blue). Hair cells fail to extend normally along the length of the cochlea, accumulating in basal and medial regions resulting in very few hair cells apically. Scale bars: 300µm. Arrowhead = extent to which hair cells are detected in the apical region, dashed lines = edge of the apical tip (and end of cochlea duct).
